# Antibiotic Resistance Awareness among Undergraduate Students in Quito, Ecuador

**DOI:** 10.3390/antibiotics11020197

**Published:** 2022-02-03

**Authors:** David Ortega-Paredes, César Marcelo Larrea-Álvarez, Lilibeth Torres-Elizalde, Sofia de Janon, Christian Vinueza-Burgos, Luis Hidalgo-Arellano, Miroslava Anna Šefcová, Gabriel Molina-Cuasapaz, Esteban Fernandez-Moreira, Marco Larrea-Álvarez

**Affiliations:** 1 Unidad de Investigación de Enfermedades Transmitidas por Alimentos y Resistencia a los Antimicrobianos (UNIETAR), Facultad de Medicina Veterinaria y Zootecnia, Universidad Central del Ecuador, Quito 170129, Ecuador; daortegap@uce.edu.ec (D.O.-P.); dsdejanon@uce.edu.ec (S.d.J.); cvinueza@uce.edu.ec (C.V.-B.); 2 Laboratorio de Referencia de *E. coli*, Department of Microbiology and Parasitology, Veterinary Faculty, University of Santiago de Compostela, 27002 Lugo, Spain; 3 Facultad de Ciencias Médicas Enrique Ortega Moreira, Carrera de Medicina, Universidad Espíritu Santo, Guayaquil 0901952, Ecuador; 4 Research Unit, Life Science Initiative (LSI), Quito 170102, Ecuador; cmla88@hotmail.com (C.M.L.-Á.); miroslava.sefcova@gmail.com (M.A.Š.); 5 School of Biological Science and Engineering, Yachay-Tech University, Hacienda San José, Urcuquí 100650, Ecuador; lilibeth.torres@yachaytech.edu.ec; 6 Facultad de Medicina Veterinaria y Zootecnia, Universidad Central del Ecuador, Quito 170129, Ecuador; lrhidalgo@uce.edu.ec; 7 Facultad de Ciencias Agropecuarias y Recursos Naturales, Carrera de Medicina Veterinaria, Universidad Técnica de Cotopaxi, Latacunga 050101, Ecuador; edie.molina7278@utc.edu.ec

**Keywords:** antibiotic resistance, Ecuador, antibiotic awareness, university students, cross-sectional survey

## Abstract

The inappropriate use of antibiotics leads to antibiotic resistance, which reduces their efficacy. The education of undergraduates is likely to influence their practices. Assessing awareness is critical in the general effort to confront the spread of antibiotic resistance. This cross-sectional investigation was carried out using the questionnaire “Antibiotic resistance: Multi-country public awareness” developed by the World Health Organization. Students from different backgrounds at the Central University participated in the study (*n* = 733). The survey comprised five sections: demographics, knowledge, usage, sources of information, and attitudes. The rate of correct answers was 64.88%; differences were detected between programs of study (*p* < 0.001); effect size analysis showed that these differences cannot be considered large. Individuals from applied sciences scored higher than their counterparts from social studies. Mostly, interviewees were knowledgeable about usage, but mistakenly associated antibiotics with conditions such as cold/flu or viral illnesses; also, they associated antibiotic resistance with the patient and not with bacteria. Despite these misconceptions, positive attitudes were registered overall, and students generally adhered to common practices. They cited doctors/nurses and teachers as sources of information. As a consequence, it is recommended to develop courses that address deficient knowledge regarding antibiotic resistance, especially for individuals affiliated to social disciplines.

## 1. Introduction

A major challenge for public health is the increment in resistance to common antimicrobial compounds. Consequently, antimicrobial resistance (AMR) is regarded as a priority, and the main goals of the World Health Organization (WHO) aim at containing this problem [1,2]. Microorganisms can develop resistance to antimicrobial agents. In bacteria, for instance, antibiotic resistance traits can arise from mutations in the genome [3], which can then be mobilized between and within DNA molecules via gene cassettes or integrons, transposons, and insertion sequences [4,5]. Furthermore, these traits can be assimilated through horizontal gene transfer, involving integrative conjugative elements and plasmids [6]. The inappropriate use as well as overuse of antibiotics are considered major factors associated with the expansion and dissemination of AMR globally [7], which could be exacerbated during the ongoing COVID-19 pandemic [8].

Low- and middle-income countries will encounter the greatest concerns associated with public health, due mainly to the prevalence of infections, availability of antibiotics, scarcity of prescribing guidelines, and limited access to low-cost diagnostics tests [9,10]; AMR is crucial in low-resource settings as it represents a potential cause of death [11]. In Ecuador, national policies and plans have been devised and executed with the goal of reducing the dissemination of antibiotic resistance [12,13]. Additionally, the safe consumption of medicines is guaranteed by the organic law for health, which specifies that they must be offered for sale only by accredited dealers. The law also stipulates that prescriptions are required and that these must be made by certified professionals, except for over-the-counter drugs [14]. In spite of these guidelines, inappropriate use of antibiotics has been reported in several Ecuadorian populations [15,16,17].

Previous research has highlighted that during undergraduate training, education on antibiotic use has a positive influence on the perspectives and practices of future professionals [18]. It has been determined that biases in such areas may influence antibiotic usage [19]. Therefore, assessing knowledge, attitudes, and practices (KAP) regarding antibiotic resistance in undergraduate students has been the goal of many investigations; most of these focused on medicine and pharmacy students [20,21,22,23,24]. These studies have aimed at identifying deficient issues, and thus generating valuable data for designing future health programs and preventive strategies accordingly. The communication of strategic information, especially the link between antibiotic misuse and AMR emergence, will prove crucial for optimizing such strategies [25,26].

In Ecuador, KAPs have been assessed in certain populations, including caretakers as well as adult and young mothers [15,27]. Two studies have so far reported a moderate level of knowledge among medical students, both undergraduate and postgraduate; attitudes and perspectives were not evaluated [24,28]. There is no data regarding antibiotic awareness in a general undergraduate population. It is important to note the importance of diverse disciplines concerning AMR. For instance, journalism students will be of prime importance as they could contribute to improve communication strategies aimed at the general public. Likewise, law students can play a crucial role in enforcing appropriate laws for containing antibiotic misuse. Recent outcomes have shown that biology-background undergraduates were more knowledgeable concerning AMR than their non-biology counterparts [20,22]. Interestingly, studies conducted among Ecuadorian undergraduates have revealed that students not associated with applied/biological sciences were not familiar with basic genetic concepts regarding inheritance or SARS-CoV-2 [29,30].

Consequently, the present study aimed at measuring KAPs with respect to antibiotic resistance in undergraduates associated with applied and social sciences in Quito, Ecuador. We utilized the questionnaire “Antibiotic resistance: Multi-country public awareness” developed by WHO, which has been successfully applied on different occasions [22,23,31].

## 2. Results

### 2.1. Demographic Data and Scores

The majority of participants were females. Individuals between 22 and 24 years of age constituted the most populous age group. Interviewees were mainly associated with programs in applied sciences (Table 1). The general mean score was 11.03 ± 2.7 out of 17, which corresponds to a correct rate of 64.88%. No differences were observed with regard to sex and age, although students enrolled in programs categorized as applied sciences scored higher than those affiliated to programs classified as social sciences. Effect size measurements suggested that these differences could be considered medium (Table 1).

### 2.2. Knowledge Regarding Antibiotics and Resistance

**Figure 1:**
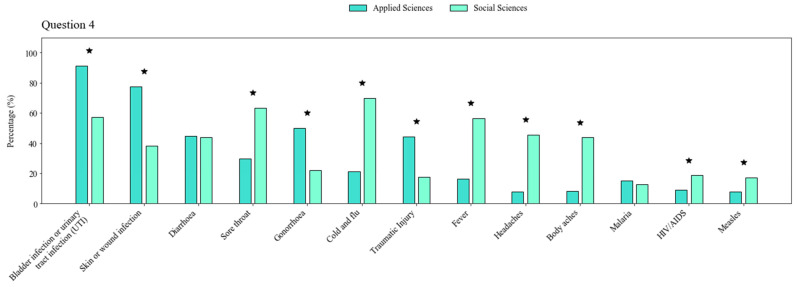
Percentages of options selected by respondents regarding disease conditions treated with antibiotics. ^⋆^ designates significant differences.

Students were aware that antibiotics must be utilized according to prescriptions (Q1) (85%, *n* = 659). Only 7% of them (*n* = 53) agreed that it is acceptable to use antibiotics given to someone else as long as they treat the same disease, although 12% (*n* = 89) chose the “Do not know” alternative (Q2). Similarly, 12% of participants (*n* = 89) selected the same option when asked about the appropriateness of buying previously used antibiotics when faced with sickness (Q3). The majority of them, however, recognized that this would not be correct (74%, *n* = 546). More than 60% of respondents associated antibiotics with UTI (urinary tract infection) and skin/wound infection; around 40% of them linked antibiotics with diarrhea, sore throat, gonorrhea, cold and flu. Around 30% of students asserted that antibacterial drugs could be employed to treat traumatic injury and fever. They also stated that head (22%) and body (21.6%) aches, malaria (14.2%), AIDS (12.7%), as well as measles (11.3%) could be treated in the same way (Q4) (Supplementary Results, Figure S1). People trained in social disciplines were in general more dubious than those enrolled in applied sciences. Bladder infection or UTI was the only correct option selected by more than 50% of students in social sciences. Furthermore, they linked treatment of sore throat, cold/flu, fever, head, and body aches with antibiotics. Particularly, students in both disciplines were uncertain about the use of these drugs against diarrhea (Figure 1).

Additional statements were used to measure knowledge on antibiotic resistance (Q5) (Table 2). Individuals were aware that infections are becoming resistant to drugs (Q5.2); similarly, they were familiar with the notion that antibiotic resistance could affect them, their families, and the overall national population (Q5.4–5). In general, students recognized that antibiotic-resistant infections could make medical procedures more dangerous (Q5.8). Conversely, participants were less confident with statements regarding the difficulty of treating infections caused by resistant bacteria (Q5.3), or the easiness with which resistant bacteria can spread among people (Q5.7). Furthermore, only over half of the population accepted that antibiotics are used in farms nationally (Q5.9). In particular, 75% of interviewees considered antibiotic resistance as a process in which the body of the patient becomes resistant to drugs (Q5.1) (Table 2). This was evident among students enrolled in programs concerning social sciences, where only 14% of participants chose the right answer. Likewise, less than 32% of individuals associated with programs in applied sciences responded accurately.

### 2.3. Usage

The outcomes showed that 34% (*n* = 252) of the students had used antibiotics in the previous month, and 31% (*n* =230) had done so in the preceding six months. Only 18% (*n* = 132), reported having taken antibiotics in the previous year (Q6). The majority of interviewees affirmed obtaining the antibiotics from a general practitioner (70%, *n* = 514) (Q7). Seventy-five percent (*n* = 558) admitted receiving instructions from a healthcare professional, whereas 25% (*n* = 187) did not or could not remember (Q8). In general, antibiotics were said to be mainly acquired in pharmacies (Q9) (92%, *n* = 676), although some people accepted receiving them from a relative/friend (2%, *n* = 16), or using stored supplies (1%, *n* = 11) (Supplementary Materials File S1).

### 2.4. Sources of Information Related to Specialized Terminology

Overall, participants affirmed that physicians and nurses were the main source of information regarding specific terms such as antibiotics and antimicrobial resistance, super bacteria, RAM, drug resistance and antibiotic resistance bacteria (Q10). Teachers and pharmacists were also mentioned, although to a lesser extent. Public campaigns and media were not cited as important (Supplementary Materials File S1).

### 2.5. Attitudes towards Antibiotics and Resistance

**Figure 2:**
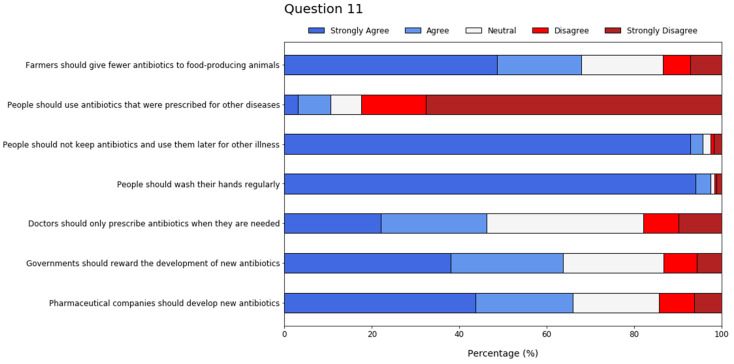
Eight-point Likert scale results from student feedback concerning potential actions that would help address antibiotic resistance.

Potential actions that would help address the problem of antibiotic resistance were presented to participants (Q11) (Figure 2). They mostly adhered to the proposed statements, although around 40% remained neutral with regard to doctors prescribing antibiotics uniquely when needed. Furthermore, more than 60% agreed with the development of novel antibiotics by governments and pharmaceutical companies.

**Figure 3:**
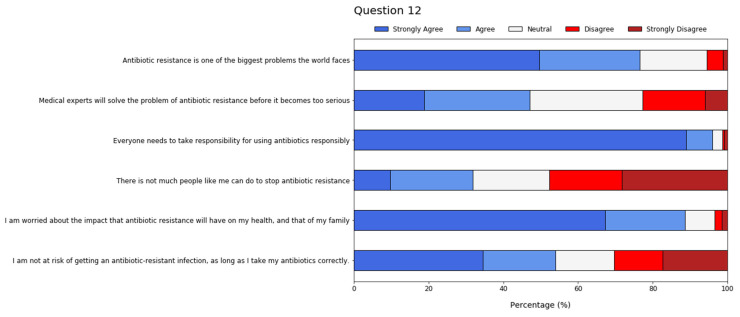
Six-point Likert scale results from student feedback regarding attitudes and perspectives about antibiotic resistance.

Additional statements were used to assess attitudes and perspectives regarding antibiotic resistance (Q12) (Figure 3). Again, most students mostly agreed with the provided options. However, around 50% of them stated they were either neutral, disagreed or strongly disagreed with the assertion concerning the role of medical experts in solving the problem of antibiotic resistance. This was also observed with regard to the risk of getting an antibiotic-resistant infection, provided that antibiotics are taken correctly. Less than 40% of interviewees agreed, or strongly agreed, with the alternative suggesting that there is not much to be done by regular people concerning this problem.

## 3. Discussion

The current survey aimed at studying undergraduate students’ knowledge, uses, sources of terminology, and attitudes with respect to antibiotic resistance. Knowledge was measured using multiple-choice and T/F questions; the overall correct rate was 64.88%. Previous studies have classified knowledge in terms of rate of correct responses as acceptable (≥80%), moderate (60–80%), and low (60%) [20,21]. Another report proposed a similar organization, although using different parameters: acceptable (≥71%), moderate (42–70%), and low (≤42%) [22]. Based on these assumptions, the measured knowledge should be considered moderate. Earlier investigations have shown similar results among undergraduate students [20,22,28]. In Ecuador, reliable studies addressing antibiotic awareness among the population are scarce. Knowledge and practices have been determined in caretakers, and in both adolescent and adult mothers [15,27]. In particular, no studies involving a general undergraduate population are available. Two surveys that were carried out among undergraduate and postgraduate medical students determined a moderate level of knowledge [24,28]. It is strongly suggested that both undergraduate and postgraduate programs include data with reference to antibiotic resistance; in this way students will be endowed with appropriate information that will more likely be helpful for improving their criteria regarding AMR. Furthermore, the present outcomes demonstrate that students enrolled in programs related to applied sciences scored higher than their colleagues affiliated to social sciences, which has been hitherto described [20,22]. Previous studies reporting knowledge of genetics and COVID-19 have shown that students not associated with applied sciences curricula have difficulties when enquired about such matters [29,30]. On the other hand, no differences were found regarding sex and age range, implying that older students are no more knowledgeable than their younger counterparts, as shown before [22,23].

Viral infections, fever, sore throat as well as head and body aches were mentioned as appropriate for being treated with antibiotics; these results are consistent with previous findings that have exhibited various misunderstandings concerning practices [22,23,32,33,34]. Some of these misconceptions have proven to be common among Ecuadorians [15,24]. Moreover, most participants considered antibiotic resistance an issue when the body of the user becomes resistant to the drugs. Importantly, these misconceptions were more popular among students in the social disciplines. A lack of understanding of the reasons why antibiotics should not be used for such conditions may be deemed responsible for the inappropriate selections. Information about antibiotic prescription and use does not seem sufficiently available or discernible to students, especially to those not familiar with scientific literature. It has been suggested that programs providing basic information about genetics or infectious diseases should be implemented as required credit courses, mainly for undergraduates associated with “social” curricula [30]. Undoubtedly, including antibiotic data in such courses will contribute to the promotion of protective health measures aimed at reducing their misuse.

Less than 40% of participants declared having used antibiotics in the preceding six to twelve months. Students mostly adhered to recommended practices such as following instructions from health care professionals, receiving professional advice, or acquiring antibiotics in pharmacies. Appropriate practices have also been observed among Ecuadorian medicine students [24]. However, incorrect use of antibiotics has been reported in studies focusing on different groups, including caretakers, farmers, or sales agents [15,16,17]. This could thus lead to a generalized misuse of antibiotics. Numerous socioeconomic factors may influence practices among citizens, predominantly in non-industrialized societies. Poor regulation, consumer demand, misinformation, or inappropriate prescription can be cited as the most critical [35,36,37,38]. In Ecuador, national plans and policies aimed at preventing and controlling the spread of AMR have been implemented [12,13]. In addition, the organic law for health indicates that in order to guarantee the safe use and consumption of medicines, they must be marketed in legally authorized establishments, and prescriptions issued by accredited professionals are required, except for over-the-counter drugs [14]. Despite these regulations, one study revealed that veterinary sales agents regularly recommended the applications of antibiotics for incorrect reasons, such as the use of an inappropriate class for a particular disease treatment or their application as animal growth promoters [16].

Proper information is fundamental for refining safe practices among people, not only regarding antibiotic use [39], but also with reference to important infectious diseases [40,41]. However, it has been argued that improving knowledge is not necessarily associated with proper practices or corrections of common misbehaviors [42,43]. Here, despite that the level of knowledge was moderate, respondents commonly adhered to recommended practices. It seems important, thus, to disclose answers at the end of the questionnaire, as hitherto suggested [30,44]. This practice could indeed be helpful in correcting or confirming popular perceptions among participants. In general, respondents asserted that in addition to doctors and nurses, teachers were also regarded as important sources of crucial terminology, which supports what has been demonstrated recently [22]. This reinforces the notion that implementing courses will increase the relationship of students with key aspects of antibiotic awareness. Notably, interviewees did not mention public campaigns and media as sources of key information. Hence, it is advised to design and execute antibiotic awareness campaigns that offer high-quality health information and make use of popular means of communication (e.g., social media). Most participants held positive attitudes towards potential actions that might help attenuate the problem of resistance. However, over half of the population considered that as long as they take their antibiotics correctly, they are not putting themselves at risk of getting an antibiotic-resistant infection, which is not the case [45]. Likewise, around half of the population either agreed or were neutral with the statement implying that there is not much common people can do to stop antibiotic resistance. These misconceptions have been documented in similar studies [1,34]. Arguably, the public may not be aware of the active role that they can play in addressing AMR. Thus, educational campaigns must place emphasis on reinforcing the concept that tackling this issue requires a concerted action between the general public, policy makers, and agronomy and health professionals.

The results described herein were comparable to those documented in studies conducted in various countries [20,22,28]. Therefore, the advocated actions, including compulsory courses, appropriately designed preventive strategies, and public health campaigns, are applicable to other regions as well, especially to those with similar socio-economic conditions. In fact, emphasis has been made on introducing courses regarding AMR in undergraduate curricula [46,47]. Likewise, the establishment of antimicrobial stewardship programs has been suggested [47]. In addition, the setting up of a network in charge of monitoring and evaluating local AMR tendencies in districts and hospitals has been recommended as a potential workable action [47]. The aim of these proposals is to increase AMR awareness and thus try to reduce its negative effects.

This research presents some limitations. Firstly, the data is not generalizable to other regions as it was carried out in Quito at the Central University; this is important as educational standards are not consistent across the country. The aim of the study was to develop a systematic understanding of antibiotic resistance awareness among undergraduate students from different backgrounds, and also to generate quantitative data that could be used for further comparisons and evaluations. Secondly, no causal inferences could be drawn due to its cross-sectional design. Finally, the actual knowledge may be overestimated as it is based on voluntary responses; thus it is recommended to use probability sampling for further research.

## 4. Materials and Methods

### 4.1. Study Design and Procedure

This cross-sectional survey was conducted from October to November 2019 amongst undergraduate students from the Central University of Ecuador in Quito, which is the oldest, largest, and most populated university in the country. Individuals were selected based on non-random criteria, as this research aimed to develop an initial understanding with respect to antibiotic awareness among university students with different backgrounds. The current study did not seek to test any hypothesis about a larger population; thus it was carried out using voluntary responses. The sample size was determined using the online calculator Raosoft [48], with a 50% response distribution, a 95% confidence level and a 5% margin of error. As the total population of the university at the moment of testing was 38,666 students [49], a sample of 381 participants was needed. However, the number of completed forms was 733, which almost doubled the estimated value. Printed surveys were distributed among students before class hours. Interviewees were provided with appropriate instructions for completing the survey, and responses were disclosed at the end of the questionnaire.

### 4.2. Study Instrument

The questionnaire “Antibiotic Resistance, Multi-country public awareness survey” [50] was developed to assess antibiotic awareness among the public and has been successfully used in several investigations [22,23,31]. Permission was obtained to reproduce the questionnaire, reference number: 382654. The form was presented in Spanish, and consisted of five sections: (I) demographics, (II) knowledge, (III) usage, (IV) sources of information, and (V) attitudes (Supplementary Materials Table S1). Demographic information included sex, program of study (applied sciences and social sciences), and age range (19–21, 22–24, ≥25). “Social sciences” included programs dedicated to the analysis of individuals and societies such as politics, linguistics, law, communication or history. On the other hand, careers associated with medicine, biology, chemistry, and engineering were considered as “Applied sciences”. All sections, except demographics, utilized multiple-choice questions. Section V included Likert scales, while section II contained true and false (T/F) questions; “do not know” responses were considered incorrect. In this section, correct answers were given 1 point for a total of 17. Scores ranged from 0 to 17, with better results implying higher knowledge.

### 4.3. Ethical Considerations

The Ethics and Bioethics Committee for Research of the Espiritu Santo University read and approved the related protocols (Reference number: Antibio 6- 2018). All procedures involving humans were performed in accordance with the Declaration of Helsinki. The questionnaire was anonymous, voluntary and the information gathered did not put the interviewees at risk in any form. Informed consent was obtained from all subjects prior to their participation (Supplementary Materials Table S2). Interviewees were able to withdraw from the session at any time.

### 4.4. Statistical Analyses

Data description was carried out using percentages and frequencies. Normality was assessed with Shapiro–Wilk’s test, and homogeneity of variance was determined by Levene’s test. For non-normally distributed and homoscedastic (program of study) as well as heteroscedastic data (age, gender), the Kruskal–Wallis test was employed. The mean is affected as a measure of the central tendency of distribution when this distribution is non-symmetrical. Hence, medians were employed as they depict more suitably the center of distribution in such conditions. Statistical significance was set at *p* < 0.05. The statistical approach based on null hypothesis significance testing does not provide information regarding the magnitude of an effect of interest (e.g., differences between demographic variables), and the precision of that estimate [51,52,53]. Consequently, effect size measures and their confidence intervals were also estimated for each test. Eta Squared (η2) was calculated for the Kruskal–Wallis test; values of 0.01, 0.06 and 0.14 denote small, medium and large differences, respectively [51,52,53,54]. For comparing frequencies in two population categories, we used the chi-square test. Analyses were performed in MATLAB^®^ version 9.9.9341360 (MathWorks, Natick, MA, USA) (R2016a); figures were rendered with Python’s plotting library, Matplotlib 3.0.3 (Python Software Foundation, Fredericksburg, VA, USA).

## 5. Conclusions

This study provides a comprehensive examination of antibiotic awareness among undergraduate students in Quito Ecuador, including individuals from different academic backgrounds. The overall correct rate was 64.88%, which should be considered moderate. Students from applied sciences scored higher than those affiliated to social disciplines. In general, respondents possessed good knowledge with regard to practices, although relevant misconceptions were detected as some erroneously recognized antibiotic use for non-associated conditions. In particular, almost three-quarters of the population stated that antibiotic resistance arises when the body of the patient becomes resistant. Students quoted doctors and nurses along with teachers as sources of key information; educational campaigns were not mentioned as relevant. Respondents showed positive attitudes and mostly adhered to common practices. These outcomes provide important insights into our current understanding of antibiotic awareness in a key part of the population, which will be certainly helpful when designing not only health programs and preventive strategies, but also educational courses aimed at limiting the spread of antimicrobial resistance.

## Figures and Tables

**Table 1:** Demographics and scores.

Variables	Number of Participants	%	Median Score (Maximum 17)	F-Value	*p*-Value	η2—Eta Squared	95% CI *	
**Sex**				0.062	0.80	−0.0012	−0.001	0.005
Female	482	65.75	10.96 (3.4)					
Male	251	34.25	10.92 (4.9)					
Age				2.48	0.28	0.0007	−0.003	0.02
19–21	55	7.50	10 (4.4)					
22–24	434	59.21	11 (3.4)					
≥25	244	33.29	10.96 (3.3)					
Program				266.54	<0.001	0.36	0.31	0.42
Applied sciences	459	62.62	12 (3)					
Social sciences	274	37.38	9.5 (3)					

Values are medians plus their corresponding interquartile range (IQR). * Confidence intervals for η2.

**Table 2:** Statements used to assess antibiotic knowledge among undergraduates.

	Correct Answers	%	Incorrect Answers	%
Q5.1 Antibiotic resistance occurs when your body becomes resistant to antibiotics and they no longer work well (F)	184	25.10	549	74.90
Q5.2 Many infections are becoming increasingly resistant to treatment with antibiotics (T)	681	92.91	52	7.09
Q5.3. If bacteria are resistant to antibiotics, it can be very difficult or impossible to treat the infections they cause (T)	569	77.63	164	22.37
Q5.4. Antibiotic resistance is an issue that could affect me or my family (T)	631	86.08	102	13.92
Q5.5. Antibiotic resistance is an issue in other countries but not in Ecuador (F)	696	94.95	37	5.05
Q5.6. Antibiotic resistance is only a problem for people who take antibiotics regularly (F)	511	69.71	222	30.29
Q5.7 Bacteria resistant to antibiotics can be spread from person to person (T)	369	50.34	364	49.66
Q5.8. Antibiotic-resistant infections could make medical procedures like surgery, organ transplants, and cancer treatment more dangerous (T)	645	87.99	88	12.01
Q5.9. In Ecuador, antibiotics are widely used in agriculture and farms (T)	428	58.39	305	41.61

## Data Availability

The data presented in this study are available on request from the corresponding authors.

## Supplementary Materials

The following are available online at https://www.mdpi.com/article/10.3390/antibiotics11020197/s1. Table S1. Survey; Table S2. Consent Information Sheet, Blank. File S1: Results and figures.
