# Supplementary material for: Antibiotic Resistance Awareness among Undergraduate Students in Quito, Ecuador
Source: Antibiotics (Basel). 2022 Feb 3;11(2):197. doi: 10.3390/antibiotics11020197 (PMC8868098; doi:10.3390/antibiotics11020197)
Supplement: Supplementary file 1 [file antibiotics-11-00197-s001.zip › Supplementary Materials, Table S2.Consent Information sheet, blank.pdf]

YACHAY-TECH UNIVERSITY CONSENT TO PARTICIPATE IN A RESEARCH STUDY

Project: Assessing antibiotic awareness in undergraduate students

Research Project Director:	Marco Larrea-Álvarez PhD., Professor/Researcher, School of Biological Science and Engineering, Yachay-Tech University, Hacienda San José, Urcuquí 100650, Ecuador. e-mail: malarrea@yachaytech.edu.ec

Study Coordinator:	David Ortega-Paredes, MSc., daortegap@gmail.com

This is a research study about assessing knowledge, attitudes and practices regarding antibiotic use in undergraduate students.

1. DETAILED STUDY INFORMATION

Please take your time to make your decision about participating. If you have any questions, you may ask the assistants.

You are being asked to take part in this study because you are currently enrolled in a study program at Universidad Central del Ecuador.

-Purpose

The aim of this study is to obtain information regarding antibiotic awareness among students of different backgrounds, including applied and biological sciences.

This project does not have any financial support.

-Population of study

The minimum required is 381 participants. However, we are aiming to interview about 800 people.

-Procedures/tests/activities

- You will answer a 5-minute questionnaire aimed at measuring antibiotic awareness; it includes x sections. X, x, x, x, and x.
- After answering the survey, the correct answers to the T/F questions will be shown.

- Study location: All these activities will be carried out at the University before class hours.

-Participation

You can decide to stop at any time. Just tell the assistant that you wish to stop being in the study.

Also, the study researcher may stop you from taking part in this study at any time if he or she believes it is in your best interest, if you do not follow the study rules, or if the study is stopped.

-Potential risks

There are no risks associated with the survey. All information will be kept confidential.

-Potential benefits

There will be no direct benefit to you from participating in this study. However, the information that you provide may help health professionals better understand/learn more about antibiotic awareness in this key population.

Use of Information

Researchers will use your answers to conduct the study. Once the study is done using your information, we may share them with other researchers so they can use them for other studies in the future. We will not share your name or any other personal information that would let the researchers know who you are. We will not ask you for additional permission to share this de-identified information.

Will information about me be kept private?

Personal information gathered for this study will be kept private. However, your personal information may be given out if required by law. If information from this study is published or presented at scientific meetings, your name and other personal information will not be used.

Authorized representatives from the following organizations may review your research data for the purpose of monitoring or managing the conduct of this study:

- Representatives of Yachay-Tech University and Universidad Central

-Contact

You can talk to the researcher(s) about any questions, concerns, or complaints you have about this study. See details at the beginning of the document.

2. CONSENT

You have been given a copy of this consent form to keep.

PARTICIPATION IN RESEARCH IS VOLUNTARY. You have the right to decline to be in this study, or to withdraw from it at any point without penalty or loss of benefits to which you are otherwise entitled.

If you wish to participate in this study, please answer the attached survey.

Thank you,

Dr. Marco Larrea-Álvarez PhD., Project Director.
Professor/Researcher, School of Biological Science and Engineering, Yachay-Tech University, Hacienda San José, Urcuquí 100650, Ecuador. e-mail: malarrea@yachaytech.edu.ec.

CONSENTIMIENTO DE LA UNIVERSIDAD YACHAY-TECH PARA PARTICIPAR EN UN ESTUDIO DE INVESTIGACIÓN

Projecto: Evaluación de la concienciación sobre antibióticos en estudiantes de pregrado

Director:	Marco Larrea-Álvarez PhD., Professor/Researcher, School of Biological Science and Engineering, Yachay-Tech University, Hacienda San José, Urcuquí 100650, Ecuador. e-mail: malarrea@yachaytech.edu.ec

Coordinador:	David Ortega-Paredes, MSc., daortegap@gmail.com

Se trata de un estudio de investigación sobre la evaluación de conocimientos, actitudes y prácticas con respecto al uso de antibióticos en estudiantes de pregrado.

1. INFORMACIÓN DETALLADA DEL ESTUDIO

Tómese su tiempo para tomar una decisión sobre su participación. Si tiene alguna pregunta, puede preguntar a los asistentes.

Se le solicita que participe en este estudio porque actualmente está inscrito en un programa de estudios en la Universidad Central del Ecuador.

-Propósito

El objetivo de este estudio es obtener información sobre el conocimiento de los antibióticos entre estudiantes de diferentes orígenes, incluidas las ciencias aplicadas y biológicas.

Este proyecto no cuenta con ningún apoyo económico.

-Población de estudio

El mínimo requerido es de 381 participantes. Sin embargo, nuestro objetivo es entrevistar a unas 800 personas.

- Procedimientos / pruebas / actividades

- Responderás a un cuestionario de 5 minutos destinado a medir el conocimiento de los antibióticos; incluye x secciones. X, x, x, x y x.

- Después de responder la encuesta, se mostrarán las respuestas correctas a las preguntas T / F.

- Lugar de estudio: Todas estas actividades se realizarán en la Universidad antes del horario de clases.

SIDAD
HAY
TECH

- Participación

Puede decidir detenerse en cualquier momento. Simplemente dígale al asistente que desea dejar de estar en el estudio.

Además, el investigador del estudio puede impedirle participar en este estudio en cualquier momento si cree que es lo mejor para usted, si no sigue las reglas del estudio o si el estudio se detiene.

-Riesgos Potenciales

No hay riesgos asociados con la encuesta. Toda la información se mantendrá confidencial.

-Beneficios Potenciales

No obtendrá ningún beneficio directo por participar en este estudio. Sin embargo, la información que proporcione puede ayudar a los profesionales de la salud a comprender mejor / aprender más sobre la concienciación sobre los antibióticos en esta población clave.

-Uso de la Información

Los investigadores utilizarán sus respuestas para realizar el estudio. Una vez que se haya realizado el estudio con su información, es posible que la compartamos con otros investigadores para que puedan usarla en otros estudios en el futuro. No compartiremos su nombre ni ninguna otra información personal que permita a los investigadores saber quién es usted. No le pediremos permiso adicional para compartir esta información anónima.

La información personal recopilada para este estudio se mantendrá privada. Sin embargo, su información personal puede ser divulgada si así lo requiere la ley. Si la información de este estudio se publica o se presenta en reuniones científicas, no se utilizará su nombre ni otra información personal.

Los representantes autorizados de las siguientes organizaciones pueden revisar los datos de su investigación con el fin de monitorear o administrar la realización de este estudio:

-Representantes de la Universidad Yachay-Tech y la Universidad Central

-Contact

Puede hablar con los investigadores sobre cualquier pregunta, inquietud o queja que tenga sobre este estudio. Consulte los detalles al principio del documento.

2. CONSENTIMIENTO

Se le ha entregado una copia de este formulario de consentimiento para que la conserve.

LA PARTICIPACIÓN EN INVESTIGACIÓN ES VOLUNTARIA. Tiene derecho a negarse a participar en este estudio o retirarse de él en cualquier momento sin penalización ni pérdida de los beneficios a los que tiene derecho.

Si desea participar en este estudio, responda la encuesta adjunta.

Gracias,

Dr. Marco Larrea-Álvarez PhD. Director del Proyecto.
Profesor / Investigador, Facultad de Ciencias Biológicas e Ingeniería, Universidad Yachay-Tech, Hacienda San José, Urcuquí 100650, Ecuador. correo electrónico: malarrea@yachaytech.edu.ec.
