# Supplementary material for: Antibiotic Resistance Awareness among Undergraduate Students in Quito, Ecuador
Source: Antibiotics (Basel). 2022 Feb 3;11(2):197. doi: 10.3390/antibiotics11020197 (PMC8868098; doi:10.3390/antibiotics11020197)
Supplement: Supplementary file 1 [file antibiotics-11-00197-s001.zip › Supplementary Results, Figures.pdf]

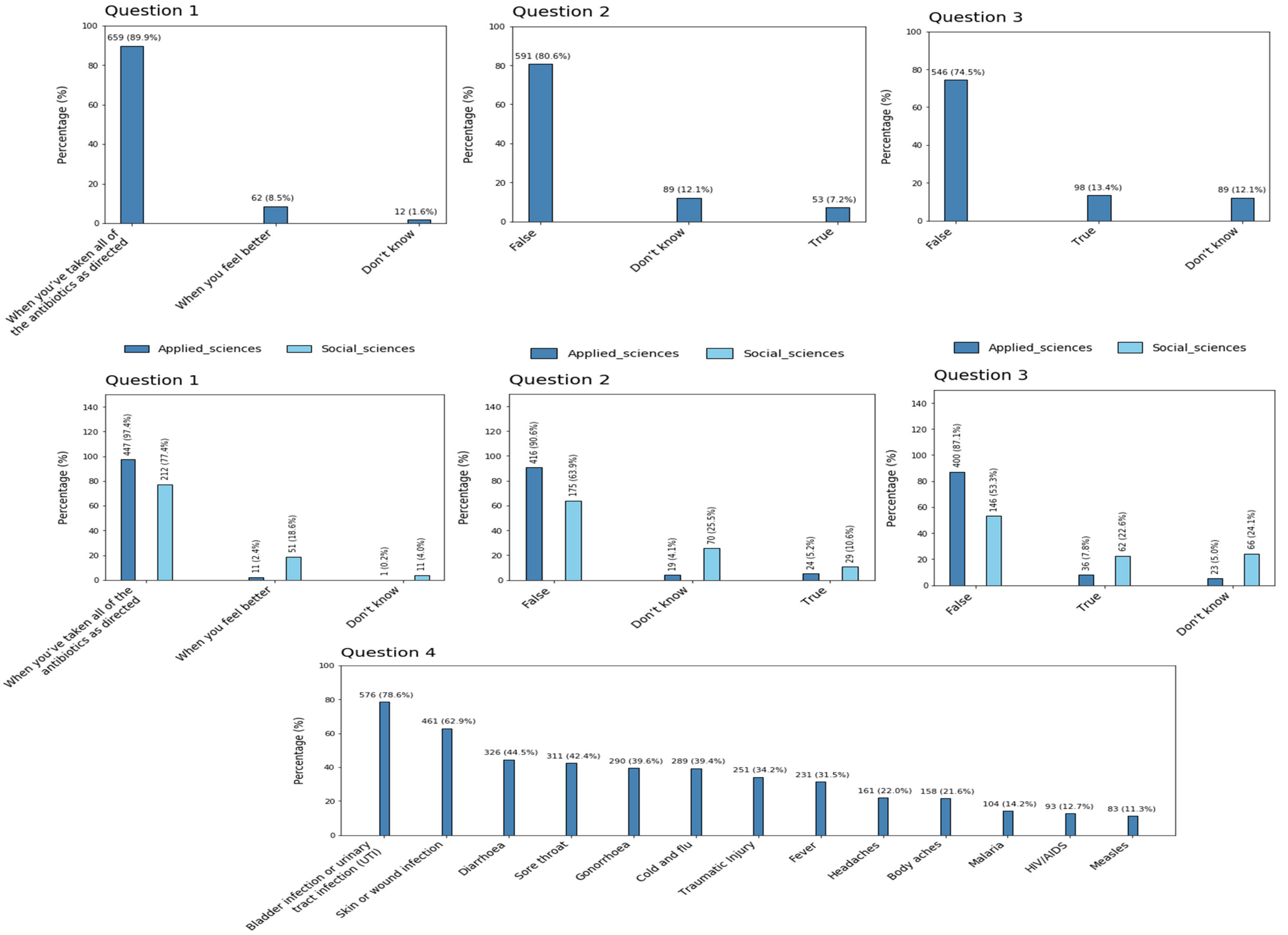

Figure S1. Percentages of selections by participants regarding knowledge of antibiotics. For Q1-Q3, the upper panels denote the total count. Questions available at Supplementary Materials, Survey.

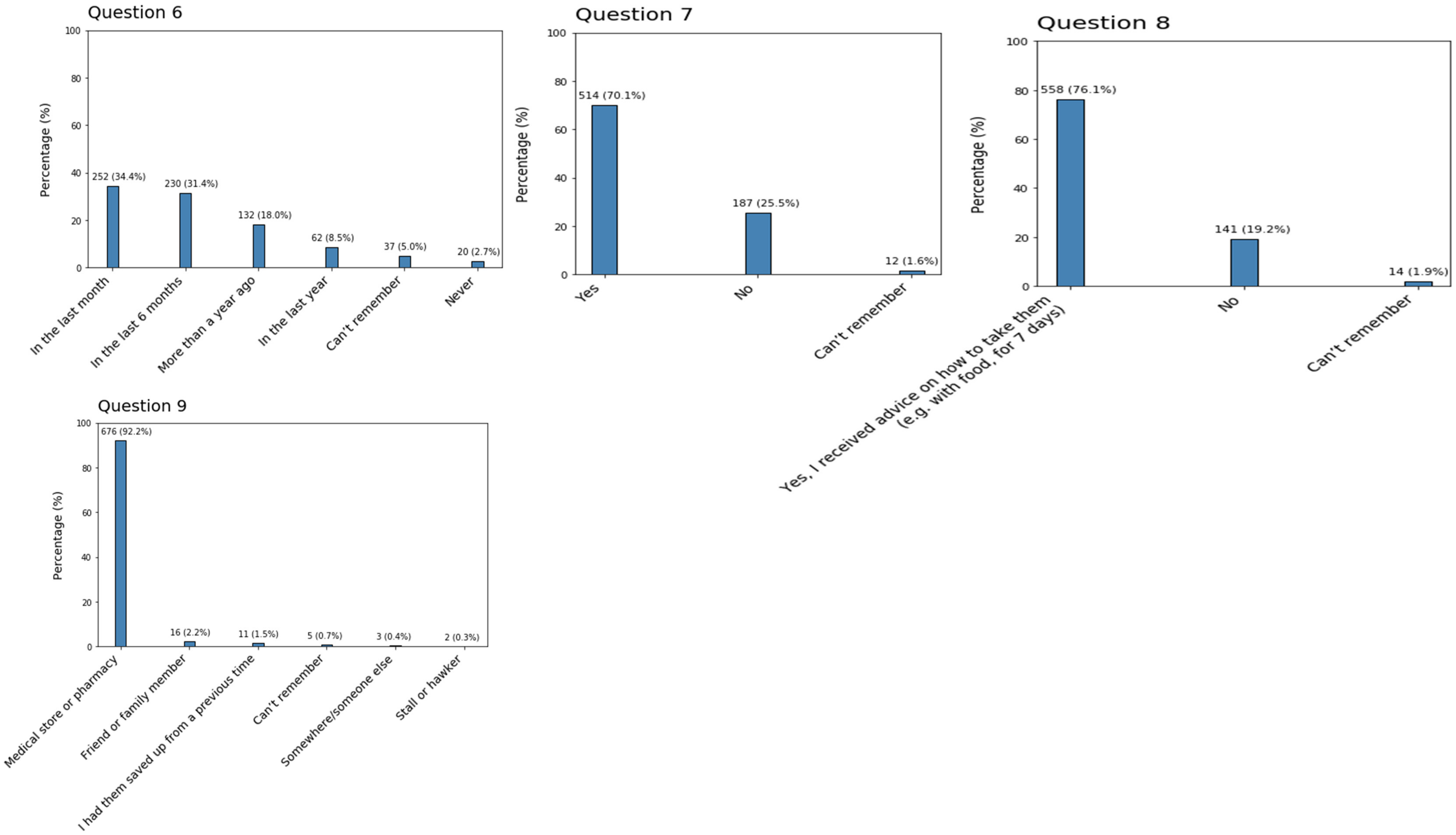

Figure S2. Percentages of selections by participants regarding knowledge use of antibiotics. Questions available at Supplementary Materials, Survey.

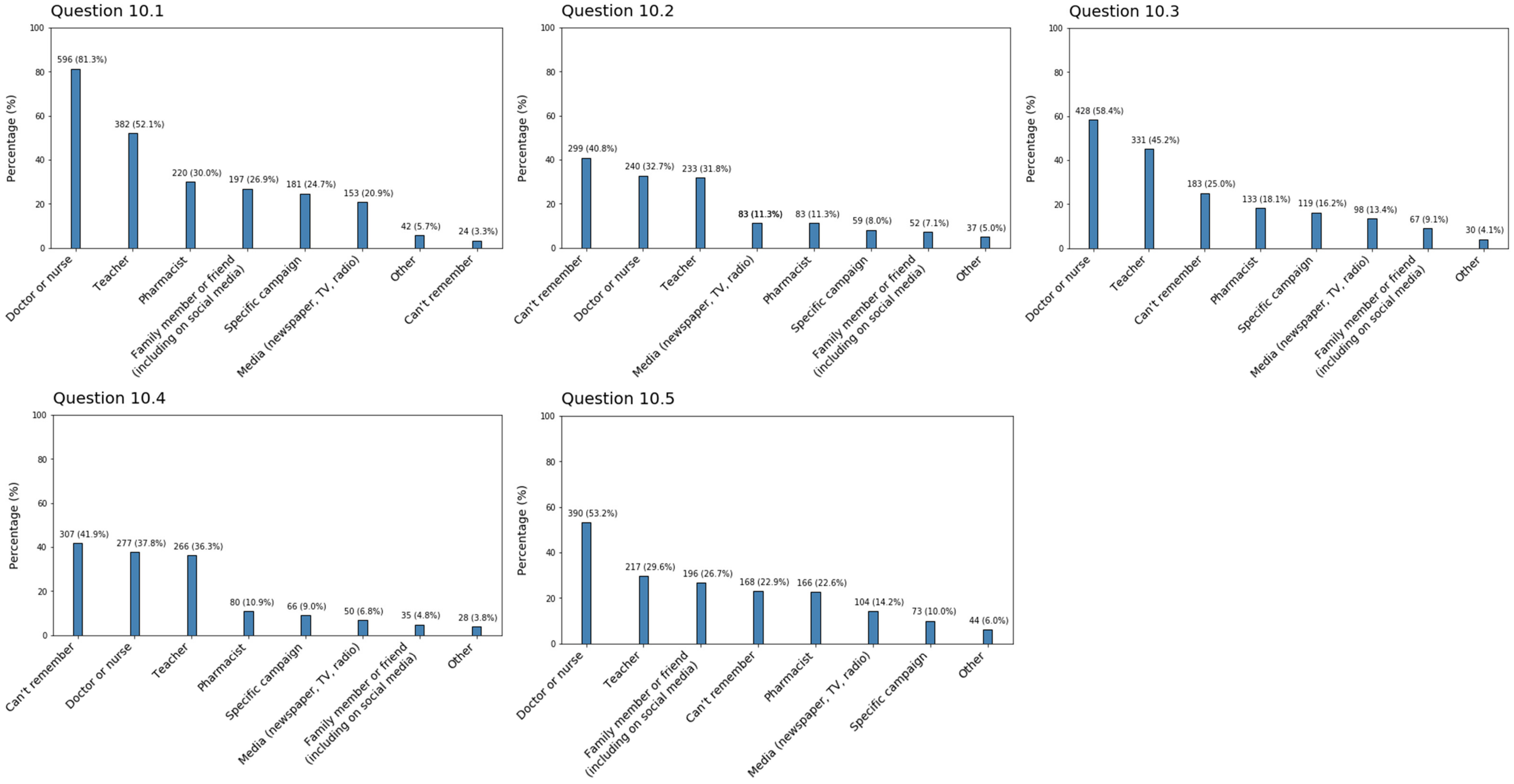

Figure S3. Percentages of selections by participants regarding sources of information of key terminology. Questions available at Supplementary Materials, Survey.
